# Computerized physician order entry of a sedation protocol is not associated with improved sedation practice or outcomes in critically ill patients

**DOI:** 10.1186/s12871-015-0161-2

**Published:** 2015-12-07

**Authors:** Samir H. Haddad, Catherine B. Gonzales, Ahmad M. Deeb, Hani M. Tamim, Abdulaziz S. AlDawood, Ibrahim Al Babtain, Brintha S. Naidu, Yaseen M. Arabi

**Affiliations:** 1 King Abdulaziz Medical City (KAMC), Riyadh, Saudi Arabia; 2 The Australian and New Zealand Intensive Care Research Centre, School of Public Health and Preventive Medicine, Monash University, Melbourne, VIC Australia; 3 King Abdullah International Medical Research Center (KAIMRC), King Abdulaziz Medical City, Riyadh, Saudi Arabia; 4 Department of Internal Medicine, Clinical Research Institute, American University of Beirut Medical Center, Beirut, Lebanon; 5 King Saud Bin Abdulaziz University for Health Sciences, Riyadh, Saudi Arabia; 6 Surgical Intensive Care Unit, Intensive Care Department, King Abdulaziz Medical City, P.O. Box 22490, Riyadh, 11426 Saudi Arabia; 7 Nursing Services, King Abdulaziz Medical City, Riyadh, Saudi Arabia; 8 Intensive Care Department, King Abdulaziz Medical City, Riyadh, Saudi Arabia; 9 Trauma & Acute Care Surgery, Department of Surgery, King Abdulaziz Medical City, Riyadh, Saudi Arabia

**Keywords:** Sedation, Protocol, Computerized physician order entry, Guidelines, Analgesics, Sedatives, Critically ill

## Abstract

**Background:**

Computerized Physician Order Entry (CPOE) analgesia-sedation protocols may improve sedation practice and patients’ outcomes. We aimed to evaluate the impact of the introduction of CPOE protocol.

**Methods:**

This was a prospective, observational cohort study of adult patients receiving mechanical ventilation, requiring intravenous infusion of analgesics and/or sedatives, and expected to stay in the intensive care unit (ICU) ≥24 h. As a quality improvement project, the study had three phases: phase 1, no protocol, July 1st to September 30th, 2010; phase 2, post implementation of CPOE protocol, October 1st to December 31st, 2010; and phase 3, revised (age, kidney and liver function adjusted) CPOE protocol, August 1st to October 31st, 2011. Multivariate analyses were performed to determine the independent predictors of mortality.

**Results:**

Two hundred seventy nine patients were included (no protocol = 91, CPOE protocol = 97, revised CPOE protocol = 91). Implementation of CPOE protocol was associated with increase of the average daily dose of fentanyl (3720 ± 3286 vs. 2647 ± 2212 mcg/day; *p* = 0.009) and decrease of hospital length of stay (40 ± 37 vs. 63 ± 85 days, *p* = 0.02). The revised CPOE protocol was associated with, compared to the CPOE protocol, a decrease of the average daily dose of fentanyl (2208 ± 2115 vs. 3720 ± 3286 mcg/day, *p* = 0.0002) and lorazepam (0 ± 0 vs. 0.06 ± 0.26 mg/day, *p* = 0.04), sedation-related complications during ICU stay (3.3 % vs. 29.9 %, p <0.0001), and ICU mortality (18 % vs. 39 %, *p* = 0.001). The impact of the revised CPOE protocol was more evident on patients aged >70 years or with severe kidney or liver impairment. Both the original CPOE protocol and the revised CPOE protocol were not independent predictors of ICU (adjusted odds ratio [aOR] = 1.85, confidence interval [CI] = 0.90–3.78; *p* = 0.09; aOR = 0.70, CI = 0.32–1.53, *p* = 0.37; respectively) or hospital mortality (aOR = 1.12, CI = 0.57–2.21, *p* = 0.74; aOR = 0.80, CI = 0.40–1.59, *p* = 0.52; respectively).

**Conclusions:**

The implementation of a CPOE analgesia-sedation protocol was not associated with improved sedation practices or patients’ outcome but with unpredicted increases of an analgesic dose. However, the revised CPOE protocol (age, kidney and liver function adjusted) was associated with improved sedation practices. This study highlights the importance of carefully evaluating the impact of changes in practice to detect unanticipated outcomes.

## Background

Sedation is a major component of the management of critically ill and mechanically ventilated patients. Several studies have suggested that sedation is often overused in patients in the intensive care unit (ICU) [1, 2]. Specific strategies to reduce excessive sedations have been recommended including the daily interruption of sedation [3] and implementation of protocol that facilitate the titration of sedatives to a sedation score [4, 5]. However, the use of these strategies in clinical practice has been inconsistent.

Clinical practice guidelines (CPGs) [6] were developed to assist practitioners in the establishment and implementation of sedation protocols appropriate to their patient population. The implementation of recommendations from these CPGs may involve changes in sedation practice. Evaluating the impact of such changes is an important quality assurance activity. It is essential to measure outcomes, and to determine whether the objectives of the practice change have been achieved.

The use of sedation protocols based on CPGs to guide analgesia and sedation therapy in the ICU is recommended [7] and has been shown to improve patient outcomes including mechanical ventilation duration, ICU and hospital length of stay (LOS), and ICU and hospital mortality [8, 9]. However, in an Australian ICU, a randomized trial provided no evidence of a substantial improvement of outcomes with the use of protocol-directed sedation compared with usual local management [10]. Moreover, the implementation of a sedation protocol may have been associated with adverse outcomes not previously reported in the literature.

Additionally, prolonged use of continuous infusions of analgesics and sedatives results in accumulation of these drugs as well as their active metabolites leading to over-sedation, greater hemodynamic instability, and prolonged mechanical ventilation and ICU stay [11]. The risk of accumulation of analgesics and sedatives is greater in patients with slow metabolism or low drug clearance such as elderly and patients with renal or hepatic dysfunction.

A computerized physician order entry (CPOE)-based analgesia-sedation protocol, derived from the CPGs [6], was developed for implementation in our ICU. As a continuous quality improvement project, the objective of this study was to evaluate whether the implementation of CPOE protocol improves sedation practice and patients’ outcomes.

## Methods

### Setting

The study was conducted in the 21-bed medical-surgical and trauma ICU in an 800-bed tertiary teaching hospital in Riyadh, Saudi Arabia. The ICU, which admits more than 1000 patients per year, is run as a closed unit 24 h a day, 7 days a week by in-house, full-time, critical care board-certified intensivists.

### Design

This was a prospective longitudinal, observational, cohort study that was conducted in three phases, each of 3-months duration. Phase 1 was prior to the implementation of a CPOE-based analgesia-sedation protocol (July 1st to September 30, 2010) (no protocol group). Phase 2 was immediately after implementation (October 1st to December 31, 2010) (CPOE protocol group). Finally, phase 3 was after revision of the protocol (August 1st to October 31, 2011) (revised CPOE protocol group), following a plan-do-study-act cycle. The ICU Quality Improvement Committee approved the protocol and the data collection process as it was considered as quality improvement project. Approval of the Research Committee of the hospital was not required as the protocol was introduced as a clinical tool and the study as a monitoring procedure to this tool. In addition, all arms of the study, the no protocol, the CPOE protocol and the revised CPOE protocol, were considered acceptable clinical practices.

### Patients

All consecutive patients receiving mechanical ventilation, who were judged by their treating team to require intravenous infusion of analgesics and/or sedatives, aged ≥18 years and expected to stay in the ICU for ≥24 h, were included in the study. Patients were excluded if they were being administered epidural analgesia, did not require sedation in the first 24 h, readmitted to the ICU within the same hospitalization, pregnant, post cardiac arrest, clinically brain dead, or with Do-Not-Resuscitate status.

### Study protocols

Prior to implementation of the CPOE protocol, the patients received analgesics and/or sedatives as per individual physician preference using CPOE and the following units: mcg/hour for fentanyl, mg/hour for morphine, propofol and midazolam, and mcg/kg/hour for dexmedetomidine.

The CPOE protocol group received analgesics and/or sedatives using a CPOE-based goal-directed, nurse-driven protocol that was derived from the available CPGs [6]. It incorporated the following analgesics and sedatives: fentanyl, dexmedetomidine (both, mcg/kg/hour), morphine, propofol, and midazolam (all three, mg/hour). The key components of this sedation protocol are: establishment of a sedation goal, use of pain and sedation scoring systems, and daily interruption of sedation when appropriate. Nurses used validated scales to assess, every 4 h, pain (Numeric Rating Scale [NRS]) [12–14] and sedation (Sedation-Agitation Scale [SAS]) [15], and titrated and tapered infusions to achieve and maintain the target pain (NRS ≤2) and sedation (SAS: 4 to 1 as required) scores. An increment or decrement of 10 % of the ongoing infusions was used to titrate or taper sedatives and/or analgesics until discontinuation of all infusions. Fentanyl was preferred for patients with unstable hemodynamic, renal or hepatic failure; propofol or dexmedetomidine were preferred if sedation was planned for ≤3 days and benzodiazepines if sedation was planned for >3 days.

As part of the quality improvement project, and following a plan-do-study-act cycle, we noticed, an unexpected, increase in the average daily dose of fentanyl after implementing the CPOE protocol. Consequently, the CPOE protocol was revised. After this revision, the patients (revised CPOE protocol group) received analgesics and/or sedatives using a goal-directed, nurse-driven; age, kidney and liver function adjusted sedation protocol; and the following units: mcg/kg/hour for fentanyl and propofol; mg/hour for morphine and midazolam; and mcg/kg/hour for dexmedetomidine; with similar key components of the protocol group. Patients were divided into three categories: age <60 years, and normal kidney and liver function (risk 1); age = 60 to 70 years, or moderate kidney or liver function impairment (risk 2); and age >70 years, or severe kidney or liver function impairment (risk 3) (Table 1). The upper limits of analgesics and sedatives doses were determined according to the risk category, being lowest in risk 3 category and lower in risk 2 category than in risk 1 category. “Modification of Diet in Renal Disease” (MDRD) was used as surrogate for kidney function, and “Model for End-Stage Liver Disease” (MELD) was used as surrogate for liver function. Normal kidney function was defined as MDRD ˃90, moderate kidney function impairment as MDRD = 30–90, and severe kidney function impairment as MDRD ˂30. Whereas, normal liver function was defined as MELD ˂8, moderate liver function impairment as MELD = 8–14, and severe liver function impairment as MELD ˃14.

**Table 1:** Definition of risk 1, risk 2 and risk 3 categories

Variable	Risk 1 category	Risk 2 category	Risk 3 category
Age, years	<60, and	60 to 70, or	>70, or
Kidney function	Normal (MDRD ˃90), and	Moderate impairment (MDRD = 30–90), or	Sever impairment (MDRD ˂30), or
Liver function	Normal (MELD ˂8)	Moderate impairment (MELD = 8–14)	Sever impairment (MELD ˃14)

*MDRD* Modification of Diet in Renal Disease (surrogate for kidney function), *MELD* Model for End-Stage Liver Disease (surrogate for liver function)

### Data collection

For the first seven ICU days, experienced dedicated nurses [4] collected the following data: total daily doses of analgesics and sedatives; NRS, SAS and Glasgow Coma Scale (GCS) scores every 4 h; and sedation-related complications during ICU stay. The nurse who collected data for an individual patient was not involved in that patient’s care. The following data, prospectively collected by a full-time data collector, were extracted from the ICU electronic database: patients’ demographics (age, gender, and body mass index [BMI]); Acute Physiology And Chronic Health Evaluation (APACHE) II [16]; organ failure indicators (GCS score, PaO_2_/FiO_2_, creatinine, bilirubin, platelets count, International Normalized Ratio [INR], and the presence of shock defined as hypotension [systolic blood pressure <90 mm Hg requiring vasopressors not including dopamine at doses of <5 mcg/kg/min]); admission categories (medical, surgical, trauma, respiratory, cardiovascular, and neurological); and severe chronic illnesses (chronic respiratory disease, chronic cardiovascular disease, chronic renal disease, chronic liver disease, and chronic immunosuppression).

### Outcome measurements

The primary outcomes were: mechanical ventilation duration, ICU and hospital LOS, and ICU and hospital mortality. Secondary outcomes were: average daily doses of analgesics and sedatives; average NRS, SAS and GCS scores; and sedation-related complications during ICU stay (agitation [SAS = 5]; very agitated [SAS = 6]; dangerous agitation [SAS = 7]; self-removal of endotracheal/tracheotomy tube, nasogastric tube, arterial/central/peripheral line, drains and urinary catheter; and CT-brain for mental status evaluation).

### Statistical analysis

Baseline characteristics were presented as number and percent for categorical variables, or mean and standard deviation for continuous ones. Comparison between the three groups (no protocol, CPOE protocol and the revised CPOE protocol) was carried out using the Chi-square test for categorical variables, whereas the independent student’s *t*-test was used for continuous variables. Stepwise logistic regression analyses were carried out to identify predictors of outcomes adjusting for chronic cardiovascular disease, chronic liver failure, APACHE II, PaO_2_/FiO_2_, and GCS. These predictors considered were those, which were found to be statistically significant, or those, which are known to be clinically significant. Odds Ratio (OR) and 95 % Confidence Intervals (CI) were reported, along with the p-value. All analyses were carried out using the Statistical Analyses Software, version 9.1. *P*-value of <0.05 was considered to indicate statistical significance.

## Results and discussion

### Baseline characteristics

Two hundred seventy nine patients were included in the study (no protocol = 91, CPOE protocol = 97, revised CPOE protocol = 91). Table 2 shows patients’ baseline characteristics of which most variables were not significantly different between the three groups. However, PaO_2_/FiO_2_ was higher in the no protocol group compared to the CPOE protocol group (238 ± 120 vs. 202 ± 103, *p* = 0.03), GCS was higher in the CPOE protocol group compared to the revised CPOE protocol group (11 ± 5 vs. 9 ± 4, *p* = 0.02), and chronic cardiovascular disease was more frequent in the no protocol and the CPOE protocol groups compared to the revised CPOE protocol group (18 % vs. 6 %, *p* = 0.01; 26 % vs. 6 %, *p* = 0.0001; respectively).

**Table 2:** Baseline characteristics

Variable	No protocol	CPOE protocol	Revised CPOE protocol	*P*-value	*P*-value	*P*-value
	(*n* = 91)	(*n* = 97)	(*n* = 91)	1 vs. 2	2 vs. 3	1 vs. 3
Age, years (mean ± SD)	50 ± 22	51 ± 20	49 ± 22	0.67	0.5	0.82
Male gender, n (%)	59 (65)	59 (61)	60 (66)	0.57	0.47	0.88
Vasopressors, n (%)	52 (59)	62 (64)	55 (60)	0.5	0.62	0.85
Sepsis, n (%)	32 (35)	37 (38)	30 (33)	0.67	0.46	0.75
BMI (mean ± SD)	29 ± 9	29 ± 7	29 ± 16	0.94	0.92	0.97
APACHE II (mean ± SD)	24 ± 9	25 ± 10	23 ± 8	0.45	0.13	0.47
Admission category						
Medical, n (%)	19 (21)	26 (27)	21 (23)	0.9641	0.54	0.57
Surgical, n (%)	29 (32)	30 (31)	25 (28)			
Trauma, n (%)	5 (6)	5 (5)	9 (10)			
Respiratory, n (%)	5 (6)	5 (5)	8 (9)			
Cardiovascular, n (%)	13 (14)	12 (13)	15 (17)			
Neurological, n (%)	20 (22)	19 (20)	13 (14)			
Organ failure indicators						
PaO_2_/FiO_2_ (mean ± SD)	238 ± 120	202 ± 103	211 ± 106	0.03	0.56	0.12
GCS (mean ± SD)	10 ± 4	11 ± 5	9 ± 4	0.45	0.02	0.11
Creatinine (mean ± SD)	120 ± 93	151 ± 159	120 ± 122	0.097	0.13	0.99
Bilirubin (mean ± SD)	64 ± 93	54 ± 66	51 ± 85	0.37	0.85	0.34
Platelets (mean ± SD)	200 ± 155	199 ± 160	215 ± 155	0.99	0.49	0.5
INR (mean ± SD)	1.6 ± 0.7	1.8 ± 1.4	1.7 ± 0.99	0.19	0.54	0.42
Severe chronic illnesses						
Chronic respiratory disease, n (%)	8 (9)	8 (8)	8 (9)	0.89	0.89	1
Chronic cardiovascular disease, n (%)	16 (18)	25 (26)	5 (6)	0.17	0.0001	0.01
Chronic renal disease, n (%)	10 (11)	7 (7)	9 (10)	0.37	0.51	0.81
Chronic liver disease, n (%)	10 (11)	13 (13)	5 (6)	0.61	0.07	0.18
Chronic immunosuppression, n (%)	10 (11)	16 (17)	13 (14)	0.27	0.68	0.50
Risk Groups						
Risk 1 patients, n (%)	24 (26)	24 (25)	35 (38)	0.96	0.12	0.21
Risk 2 patients, n (%)	18 (20)	19 (20)	16 (18)			
Risk 3 patients, n (%)	49 (54)	54 (56)	40 (44)			

1: No protocol; 2: CPOE Protocol; 3: Revised CPOE Protocol; *APACHE* Acute Physiology and Chronic Health Evaluation, *BMI* body mass index, *GCS* Glasgow Coma Scale, *INR* International Normalized Ratio; Risk 1: age ˂60 years, normal kidney function (MDRD ˃90), normal liver function (MELD ˂8); Risk 2: age = 60–70 years, moderate kidney function impairment (MDRD = 30–90), moderate liver function impairment (MELD = 8–14); Risk 3: age ˃70 years, severe kidney function impairment (MDRD ˂30), severe liver function impairment (MELD ˃14)

### Outcomes

The results of crude analyses of primary and secondary outcomes are shown in Table 3.

**Table 3:** Primary and secondary outcomes

Variable	No protocol	CPOE protocol	Revised CPOE protocol	*P*-value	*P*-value	*P*-value
(Mean ± SD)	(*n* = 91)	(*n* = 97)	(*n* = 91)	1 vs. 2	2 vs. 3	1 vs. 3
Primary outcomes						
Mechanical ventilation duration, mean ± SD, days	10 ± 9	12 ± 11	10 ± 8	0.21	0.2	0.98
ICU LOS, mean ± SD, days	12 ± 10	13 ± 12	13 ± 11	0.78	0.91	0.69
Hospital LOS, mean ± SD, days	63 ± 85	40 ± 37	46 ± 42	0.02	0.32	0.09
ICU mortality, n (%)	24 (26)	38 (39)	16 (18)	0.06	0.001	0.15
Hospital mortality, n (%)	36 (40)	43 (44)	29 (32)	0.51	0.08	0.28
Secondary outcomes						
Average daily doses of analgesics and sedatives						
Fentanyl, mcg/day	2647 ± 2212	3720 ± 3286	2208 ± 2115	0.009	0.0002	0.17
Morphine, mg/day	0.71 ± 4.88	0.36 ± 2.11	0.45 ± 2.18	0.54	0.79	0.64
Dexmedetomidine, mcg/day	47.6 ± 113.7	48.3 ± 153.5	40.6 ± 124.1	0.97	0.71	0.69
Midazolam, mg/day	84 ± 107	78 ± 101	68 ± 96	0.71	0.48	0.3
Propofol, mg/day	271 ± 650	274 ± 833	509 ± 961	0.97	0.07	0.052
Lorazepam, mg/day	0.09 ± 0.32	0.06 ± 0.26	0 ± 0	0.47	0.04	0.01
Haloperidol, mg/day	0.27 ± 0.87	0.42 ± 1.77	0.23 ± 0.85	0.45	0.34	0.72
Paralytics, mg/day	28 ± 61	20 ± 60	21 ± 49	0.34	0.84	0.41
Average NRS, SAS and GCS scores						
NRS score, mean ± SD	0.24 ± 0.5	0.16 ± 0.36	1.02 ± 0.73	0.20	<0.0001	<0.0001
SAS score, mean ± SD	2.5 ± 1.1	2.3 ± 1.1	2.5 ± 1.2	0.47	0.25	0.66
GCS score, mean ± SD	6.8 ± 3.2	6.6 ± 3.2	6.9 ± 2.9	0.84	0.36	0.47
Sedation-related complications during ICU stay						
Agitated (SAS = 5), n (%)	37 (40.7)	29 (29.9)	3 (3.3)	0.12	<0.0001	<0.0001
Very agitated (SAS = 6), n (%)	10 (11)	6 (6.2)	3 (3.3)	0.24	0.5	0.04
Dangerous agitation (SAS = 7), n (%)	4 (4.4)	1 (1)	2 (2.2)	0.2	0.61	0.68
NGT self-removal, n (%)	2 (2.2)	2 (2.1)	0 (0)	1	0.5	0.5
ETT self-removal, n (%)	2 (2.2)	0 (0)	0 (0)	0.23	-	0.5
Brain CT scans for mental status assessment, n (%)	1 (1.1)	0 (0)	0 (0)	0.48	-	1

1: No protocol; 2: CPOE Protocol; 3: Revised CPOE Protocol; *CT* computed tomography, *ETT* endotracheal tube, *GCS* Glasgow Coma Scale, *ICU* intensive care unit, *LOS* length of stay, *NGT* nasogastric tube, *NRS* Numeric Rating Scale, *SAS* Sedation-Agitation Scale, *SD* standard deviation

### Outcomes stratified by risk

Table 4 summarizes primary and secondary outcomes of patients with risk 1, risk 2 and risk 3 for the three study groups.

**Table 4:** Primary and secondary outcomes stratified by risks

Variable	No protocol	CPOE protocol	Revised CPOE protocol	*P*-Value	*P*-Value	*P*-Value
	(*n* = 91)	(*n* = 97)	(*n* = 91)	1 vs. 2	2 vs. 3	1 vs. 3
Risk 1 patients: age ˂60 years, normal kidney function (MDRD ˃90), normal liver function (MELD ˂8)						
Primary outcomes						
Mechanical ventilation duration, mean ± SD, days	5.9 ± 6.4	12.2 ± 12.2	10.5 ± 9.4	0.21	0.55	0.40
ICU LOS, mean ± SD, days	10.3 ± 7.4	13.3 ± 15.2	12.2 ± 11.5	0.39	0.75	0.44
Hospital LOS, mean ± SD, days	78.4 ± 95.4	52.3 ± 53.5	50.8 ± 56.6	0.25	0.92	0.22
ICU mortality, n (%)	3 (13)	3 (13)	4 (11)	1	1	1
Hospital mortality, n (%)	5 (21)	5 (21)	4 (11)	1	0.46	0.46
Secondary outcomes						
Average daily doses of analgesics and sedatives						
Fentanyl, mean ± SD, mcg/day	3694 ± 2315	3711 ± 2879	2613 ± 2257	0.98	0.11	0.08
Morphine, mg/day	2.2 ± 9.3	0.93 ± 3.8	1.09 ± 3.4	0.54	0.87	0.58
Dexmedetomidine, mean ± SD mcg/day	52 ± 112	125 ± 269	42 ± 93	0.23	0.16	0.72
Midazolam, mean ± SD, mg/day	158 ± 148	111 ± 132	98 ± 113	0.26	0.67	0.08
Propofol, mean ± SD, mg/day	564 ± 947	812 ± 1517	643 ± 1063	0.50	0.62	0.77
Lorazepam, mg/day	0.17 ± 0.52	0.10 ± 0.31	0 ± 0	0.58	-	-
Haloperidol, mg/day	0.17 ± 0.49	0.90 ± 2.8	0.37 ± 1.19	0.22	0.39	0.37
Paralytics, mg/day	50.3 ± 85.4	47.3 ± 104.2	30.5 ± 59.7	0.91	0.48	0.30
Average NRS, SAS and GCS scores						
NRS score, mean ± SD	0.28 ± 0.54	0.22 ± 0.4	1.01 ± 0.86	0.63	<0.0001	0.0002
SAS Score, mean ± SD	2.2 ± 1.1	2.5 ± 1.1	2.5 ± 1.3	0.47	0.96	0.46
GCS score, mean ± SD	6.4 ± 3.4	6.9 ± 3.4	7 ± 3.3	0.58	0.95	0.50
Sedation-related complications during ICU stay						
Agitated (SAS = 5), n (%)	7 (29.2)	9 (37.5)	0 (0)	0.54	<0.0001	0.001
Very agitated (SAS = 6), n (%)	3 (12.5)	2 (8.3)	1 (2.9)	1.00	0.56	0.29
Dangerous agitation (SAS = 7), n (%)	2 (8.3)	1 (4.2)	0 (0)	100	0.41	0.16
NGT self-removal, n (%)	1 (4.2)	0 (0)	0 (0)	1.00	-	0.41
ETT self-removal, n (%)	0 (0)	0 (0)	0 (0)	-	-	-
Brain CT scans for mental status assessment, n (%)	0 (0)	0 (0)	0 (0)	-	-	-
Risk 2 patients: age = 60–70 years, moderate kidney function impairment (MDRD = 30–90), moderate liver function impairment (MELD = 8–14)						
Primary outcomes						
Mechanical ventilation duration, mean ± SD, days	9.6 ± 8.2	12.7 ± 13.8	8.4 ± 6.0	0.40	0.22	0.64
ICU LOS, mean ± SD, days	11.3 ± 8.6	13 ± 12.1	11.4 ± 12.3	0.64	0.72	0.97
Hospital LOS, mean ± SD, days	70.8 ± 95.3	45.3 ± 27.6	53.3 ± 42.2	0.29	0.51	0.49
ICU mortality, n (%)	2 (11)	2 (11)	2 (13)	1	1	1
Hospital mortality, n (%)	3 (17)	2 (11)	3 (19)	0.66	0.64	0.87
Secondary outcomes						
Average daily doses of analgesics and sedatives						
Fentanyl, mean ± SD mcg/day	2336 ± 1979	4153 ± 3967	2759 ± 2455	0.09	0.23	0.58
Morphine, mg/day	0.19 ± 0.59	0.02 ± 0.07	0.15 ± 0.42	0.23	0.22	0.83
Dexmedetomidine, mean ± SD mcg/day	47 ± 67	54 ± 119	60 ± 214	0.82	0.92	0.81
Midazolam, mean ± SD mg/day	59 ± 75	91 ± 88	98 ± 122	0.24	0.85	0.26
Propofol, mean ± SD mg/day	201 ± 326	118 ± 285	759 ± 1425	0.42	0.1	0.14
Lorazepam, mg/day	0.75 ± 0.23	0.02 ± 0.07	0 ± 0	0.36	-	-
Haloperidol, mg/day	0.92 ± 1.58	0.26 ± 0.61	0.14 ± 0.56	0.11	0.55	0.06
Paralytics, mg/day	6.0 ± 14.4	5.4 ± 14.6	25.9 ± 57.7	0.90	0.19	0.20
Average NRS, SAS and GCS scores						
NRS score, mean ± SD	0.44 ± 0.80	0.23 ± 0.53	0.93 ± 0.69	0.35	0.002	0.06
SAS score, mean ± SD	3.1 ± 0.9	2.8 ± 1.0	2.3 ± 1.2	0.42	0.24	0.055
GCS score, mean ± SD	7.7 ± 2.2	8.2 ± 4.0	6.4 ± 3	0.64	0.13	0.14
Sedation-related complications during ICU stay						
Agitated (SAS = 5), n (%)	10 (55.6)	9 (47.4)	0 (0)	0.62	0.001	0.0004
Very agitated (SAS = 6), n (%)	3 (16.7)	1 (5.3)	0 (0)	0.34	1.00	0.23
Dangerous agitation (SAS = 7), n (%)	1 (5.6)	0 (0)	0 (0)	0.49	-	1.00
NGT self-removal, n (%)	1 (5.6)	2 (10.5)	0 (0)	1.00	0.49	1.00
ETT self-removal, n (%)	0 (0)	0 (0)	0 (0)	-	-	-
Brain CT scans for mental status assessment, n (%)	0 (0)	0 (0)	0 (0)	-	-	-
Risk 3 patients: age ˃70 years, severe kidney function impairment (MDRD ˂30), severe liver function impairment (MELD ˃14)						
Primary outcomes						
Mechanical ventilation duration, mean ± SD, days	11.3 ± 10.0	11.8 ± 9.0	10.9 ± 7.1	0.81	0.61	0.82
ICU LOS, mean ± SD, days	13.2 ± 11.6	12 ± 9.9	13.6 ± 9.5	0.56	0.42	0.86
Hospital LOS, mean ± SD, days	52.5 ± 75.4	33 ± 29.8	39.1 ± 22.7	0.10	0.28	0.25
ICU mortality, n (%)	19 (39)	33 (61)	10 (25)	0.02	0.0005	0.17
Hospital mortality, n (%)	28 (57)	36 (67)	22 (55)	0.32	0.25	0.84
Secondary outcomes						
Average daily doses of analgesics and sedatives						
Fentanyl, mean ± SD mcg/day	2232 ± 2108	3571 ± 3245	1633 ± 1719	0.01	0.0003	0.15
Morphine, mg/day	0.14 ± 0.82	0.23 ± 1.3	0 ± 0	0.68	0.19	0.24
Dexmedetomidine, mean ± SD mcg/day	46 ± 130	12 ± 51	32 ± 102	0.10	0.28	0.57
Midazolam, mean ± SD mg/day	55 ± 71	58 ± 84	30 ± 42	0.85	0.03	0.04
Propofol, mean ± SD mg/day	148 ± 508	90 ± 240	293 ± 523	0.48	0.03	0.19
Lorazepam, mg/day	0.05 ± 0.18	0.05 ± 0.28	0 ± 0	0.99	-	-
Haloperidol, mg/day	0.08 ± 0.43	0.27 ± 1.41	0.13 ± 0.51	0.34	0.51	0.58
Paralytics, mg/day	25.8 ± 54.8	12.8 ± 35.8	12.0 ± 32.6	0.17	0.91	0.15
Average NRS, SAS and GCS scores						
NRS score, mean ± SD	0.15 ± 0.27	0.11 ± 0.25	1.07 ± 0.61	0.46	<0.0001	<0.0001
SAS score, mean ± SD	2.3 ± 1.1	2.1 ± 1.0	2.6 ± 1.0	0.31	0.02	0.18
GCS score, mean ± SD	6.5 ± 3.2	5.9 ± 2.9	7.4 ± 2.6	0.32	0.01	0.17
Sedation-related complications during ICU stay						
Agitated (SAS = 5), n (%)	20 (40.8)	11 (20.4)	3 (7.5)	0.02	0.08	0.0004
Very agitated (SAS = 6), n (%)	4 (8.2)	3 (5.6)	2 (5)	0.71	1.00	0.69
Dangerous agitation (SAS = 7), n (%)	1 (2)	0 (0)	2 (5)	0.48	0.18	0.59
NGT self-removal, n (%)	0 (0)	0 (0)	0 (0)	-	-	-
ETT self-removal, n (%)	2 (4.1)	0 (0)	0 (0)	0.22	-	0.50
Brain CT scans for mental status assessment, n (%)	1 (2)	0 (0)	0 (0)	0.48	-	1.00

1: No protocol; 2: CPOE Protocol; 3: Revised CPOE Protocol; *CT* computed tomography, *ETT* endotracheal tube, *GCS* Glasgow Coma Scale, *ICU* intensive care unit, *LOS* length of stay, *MDRD* Modification of Diet in Renal Disease (surrogate for kidney function), *MELD* Model for End-Stage Liver Disease (surrogate for liver function), *NGT* nasogastric tube, *NRS* Numeric Rating Scale, *SAS* Sedation-Agitation Scale

### Predictors of ICU and hospital mortality

Multivariate logistic regression analyses showed that the CPOE protocol and the revised CPOE protocol were not independently associated with ICU (adjusted odds ratio [aOR] = 1.85, 95 % confidence interval [CI] = 0.90–3.78, *p* = 0.09; aOR = 0.70, CI = 0.32–1.53, *p* = 0.37; respectively) or hospital (aOR = 1.12, CI = 0.57–2.21, *p* = 0.74; aOR = 0.80, CI = 0.40–1.59, *p* = 0.52; respectively) mortality (Table 5).

**Table 5:** Predictors of ICU and hospital mortality (multivariate logistic regression analysis)

Variable	Adjusted odds ratio (95 % CI)	*P*-value
ICU mortality		
CPOE protocol	1.85 (0.90–3.78)	0.09
Revised CPOE protocol	0.70 (0.32–1.53)	0.37
Chronic liver disease	5.92 (2.30–15.23)	0.0002
APACHE II	1.12 (1.07–1.16)	<0.0001
Hospital mortality		
CPOE protocol	1.12 (0.57–2.21)	0.74
Revised CPOE protocol	0.80 (0.40–1.59)	0.52
Chronic liver disease	4.68 (1.70–12.89)	0.003
APACHE II	1.14 (1.09–1.18)	<0.0001

*APACHE* Acute Physiology and Chronic Health Evaluation, *CPOE* computerized physician order entry, *ICU* intensive care unit

### Predictors of ICU and hospital mortality stratified by risk

The results of multivariate logistic regression analyses for the three risk groups are shown in Table 6. In patients with risk 1 and risk 2, the CPOE protocol and the revised CPOE protocol were not independently associated with ICU or hospital mortality. In patients with risk 3, the CPOE protocol was independently associated with increased ICU mortality (aOR = 2.64, CI = 1.03–6.56, *p* = 0.04), but was not associated with hospital mortality (aOR = 1.43, CI = 0.59–3.46, *p* = 0.43). The revised CPOE protocol was not associated with ICU (aOR = 0.68, CI = 0.24–1.88, *p* = 0.45) or hospital (aOR = 1.11, CI = 0.44–2.79, *p* = 0.82) mortality.

**Table 6:** Predictors of ICU and hospital mortality stratified by risk (multivariate logistic regression analysis)

Variable	Adjusted odds ratio (95 % CI)	*P*-value
Risk 1: age ˂60 years, normal kidney function (MDRD ˃90), normal liver function (MELD ˂8)		
ICU mortality		
CPOE protocol	0.82 (0.12–5.63)	0.84
Revised CPOE protocol	0.45 (0.07–2.99)	0.41
APACHE II	1.22 (1.07–1.38)	0.002
Hospital mortality		
CPOE protocol	0.67 (0.13–3.48)	0.63
Revised CPOE protocol	0.21 (0.04–1.16)	0.07
APACHE II	1.13 (1.00–1.28)	0.0492
GCS	0.87 (0.73–1.03)	0.11
Risk 2: age = 60–70 years, moderate kidney function impairment (MDRD = 30–90), moderate liver function impairment (MELD = 8–14)		
ICU mortality		
CPOE protocol	0.54 (0.06–4.82)	0.58
Revised CPOE protocol	1.43 (0.15–13.88)	0.76
GCS	1.47 (0.99–2.18)	0.06
Hospital mortality		
CPOE protocol	0.59 (0.09–4.01)	0.59
Revised CPOE protocol	1.15 (0.198–6.74)	0.87
Risk 3: age ˃70 years, severe kidney function impairment (MDRD ˂30), severe liver function impairment (MELD ˃14)		
ICU mortality		
CPOE protocol	2.64 (1.03–6.56)	0.04
Revised CPOE protocol	0.68 (0.24–1.88)	0.45
Chronic cardiovascular disease	2.07 (0.81–5.30)	0.13
Chronic liver disease	5.87 (1.99–17.27)	0.001
APACHE II	1.11 (1.05–1.18)	0.0002
Hospital mortality		
CPOE protocol	1.43 (0.59–3.46)	0.43
Revised CPOE protocol	1.11 (0.44–2.79)	0.82
Chronic liver disease	3.25 (1.07–9.84)	0.04
APACHE II	1.12 (1.06–1.19)	<0.0001

*APACHE* Acute Physiology and Chronic Health Evaluation, *CPOE* computerized physician order entry, *ICU* intensive care unit, *GCS* Glasgow Coma Scale, *MDRD* Modification of Diet in Renal Disease (surrogate for kidney function), *MELD* Model for End-Stage Liver Disease (surrogate for liver function)

### Discussion

Our study demonstrates that the implementation of a CPOE-based goal-directed, nurse-driven, analgesia-sedation protocol was not associated with improved patients’ outcomes. We observed a significant increase in the average daily dose of fentanyl that was predominantly in patients with risk 3 (Tables 3, 4). Furthermore, the CPOE protocol was associated with a significant increase in the ICU mortality in patients with risk 3 (Table 4). However, revision of the protocol by adjusting for age, kidney and liver function resulted in elimination of the increase in the average daily dose of fentanyl, and loss of the association with mortality in patients with risk 3. The impact of the revised CPOE protocol was more evident on patients with risk 3. Compared to no protocol and to CPOE protocol, the revised CPOE protocol was associated with a significant increase in the NRS score, however, within acceptable level; and a significant decrease in the sedation-related complications during ICU stay (agitated, SAS = 5).

Optimizing sedation practice, using ICU protocols for sedation, is recognized as quality indicator and is believed to be important in improving patient outcomes [17]. However, the implementation of a sedation protocol is a dynamic process that requires reassessment. This assessment allows periodic modification of the protocol to ensure that the projects’ objectives are being met. Our study demonstrates the importance of monitoring and measuring the impact of implementation of an analgesia-sedation protocol that derived from CPGs, since paradoxical effects resulted from its implementation. In our institution, the implementation of a CPOE-based goal-directed, nurse-driven, analgesia-sedation protocol was associated with a significant increase in the ICU mortality in elderly patients (>70 years) and in patients with severe kidney or liver function impairments, and a significant increase in the average daily dose of fentanyl. The significant increase of the average daily dose of fentanyl in the CPOE protocol group compared to the no protocol group is possibly due the change of units used for dosing. Prior to implementation of the protocol; commonly and regardless of age, weight, gender, or renal or liver function; the ordered dose of fentanyl ranged from 50 to 200 mcg/hour. In the CPOE protocol, the fentanyl dose was 0.5 – 5 mcg/kg/hour. This resulted in an hourly dose that may have reached up to 500 mcg in a 100 kg patient and 700 mcg in a 140 kg patient. The trend towards higher ICU mortality in the protocol group may be potentially due to the significantly high dose of fentanyl, and differences in baseline characteristics (the CPOE protocol group had higher mean creatinine and significantly lower mean of PaO_2_/FiO_2_ ratio. The increase in the daily dose of propofol that was associated with the implementation of the revised CPOE protocol was possibly due to the decrease of the average daily dose of fentanyl that was used not only as an analgesic but also as a sedative.

Although, CPOE has been recommended [18] to reduce human and medication errors during health care delivery, and therefore improve patient safety, studies demonstrate conflicting results of its impact on morbidity and mortality [19, 20]. Han et al., documented an increase in mortality after implementation of CPOE in a tertiary care children’s hospital [19], however, Del Beccaro et al., reported that implementation of CPOE was not associated with increased mortality in tertiary care pediatric ICU [20]. Our study is the first to evaluate the impact of a CPOE-based analgesia-sedation protocol on doses of analgesics and sedatives, and on patients’ outcome. Unexpectedly, the initial implementation of CPOE-based analgesia-sedation protocol was not associated with improved outcome, however, with increased doses of analgesics, and with increased ICU mortality in patients with high-risk (risk 3).

This study has a number of strengths including the prospective nature of data collection by a full-time dedicated data collector and dedicated nurses; the collection of pharmacologic, physiologic and clinical endpoints; and the inclusion of all consecutive eligible patients during the study periods. The study also had several limitations. First, the study was observational and of pre-post design, therefore, the influence of other confounders cannot be excluded. Second, the study was conducted in a single center. Third, the use of MELD and MDRD as surrogates for kidney and liver dysfunction, respectively, is controversial. Forth, delirium, an important outcome of sedation, was not assessed during the study. Finally, the compliance with the protocols was not measured, and the use of protocol was not forced-function as drugs that are used in the protocols could be prescribed through the search function outside the protocols.

## Conclusions

The implementation of a CPOE-based analgesia-sedation protocol was not associated with improved patients’ outcome. However, its initial implementation was associated with increased ICU mortality in the elderly and in patients with severe renal or hepatic failure. Once revised and with the incorporation of age, kidney and liver function, the protocol was associated with improved sedation practices and patients’ outcomes. Our experience demonstrates that the implementation of sedation protocols should be regularly monitored to detect inadvertent consequences. When designing a sedation protocol, we suggest that it includes adjustment for patient’s age, and kidney and liver function. Integrating such sedation protocols may decrease the amount of analgesic and/or sedative agents and improve patient outcome.

## Abbreviations

APACHE: Acute Physiology and Chronic Health Evaluation
aOR: adjusted odds ratio
BMI: body mass index
CPGs: clinical practice guidelines
CPOE: computerized physician order entry
CI: confidence intervals
GCS: Glasgow Coma Scale
ICU: intensive care unit
INR: International Normalized Ratio
LOS: length of stay
MELD: Model for End-Stage Liver Disease
MDRD: Modification of Diet in Renal Disease
NRS: Numeric Rating Scale
OR: odds ratio
SAS: Sedation-Agitation Scale
